# Old age psychiatry services in the UK responding to COVID-19

**DOI:** 10.1017/S1041610220001015

**Published:** 2020-05-29

**Authors:** Krishnaveni Vedavanam, Dawne Garrett, Nathan Davies, Kirsten J. Moore

**Affiliations:** 1 Department of Cognition and Mental Health in Aging, Springfield University Hospital, London, UK; 2 Nursing Department, Royal College of Nursing, London, UK; 3 Marie Curie Palliative Care Research Department, Division of Psychiatry, UCL, London, UK; 4 Centre for Ageing Population Studies, Research Department of Primary Care and Population Health, UCL, London, UKEmail: Krishnaveni.Vedavanam@swlstg.nhs.uk

The coronavirus disease 2019 (COVID-19) pandemic has presented a profound global challenge. It has affected all areas of society on an international level. Yet, there are certain groups who are significantly more vulnerable, including older people (Verity *et al.*, 2020), those with chronic long-term conditions associated with immunosuppression, and those with mental illness. Older people with complex mental health problems, in particular, need special consideration (Yao *et al.*, 2020). They form the majority of the population of UK care homes (Prince *et al.*, 2014) and are the demographic of society worst affected by the morbidity and mortality associated with COVID-19 and are of course the patients supported by older adult mental health services. This commentary will focus on the challenges met by old age psychiatrists in the United Kingdom during the pandemic.

Older people with mental illness have significant functional and cognitive impairments, often in conjunction with multiple comorbidities and frailty (Gulpers *et al.*, 2016; Soysal *et al.*, 2017). In addition, with age comes increasing risk of social isolation and lack of disposable income (Freedman and Nicolle, 2020) which cushions other age groups from the hardships associated with quarantine. In England, we have a paradigm of inclusion for people living with dementia and talk about “living well with dementia”. However, people living with dementia are more likely to experience loneliness than the general population (Kane and Cook, 2013). Social isolation can increase the likelihood of early death by 26% (Holt-Lunstad *et al.*, 2015) and is associated with reduced cognitive function (Shankar *et al.*, 2013). Social prescribing is a national plan to increase societal connectedness to address social determinants of health such as loneliness (NHS England, 2019). Most social prescribing schemes involve a variety of community and group-based activities typically provided by voluntary and community sector organizations and supporting people living with dementia (The King’s Fund, 2017). The pandemic has required vulnerable people to be confined to their homes with no social contact which has obvious implications on socially isolating frail older people.

In addition to loneliness, the pandemic may also increase the risk of abuse, financial scams, self-neglect, fear, and distress for older people with dementia and without support (Clarke, 2020). Some people with dementia may have difficulty understanding complex instructions about self-isolation or handwashing and usual patterns of behavior such as purposeful walking or shopping may leave people open to hostility and sanction if they fail to observe social distancing, respiratory etiquette, or handwashing.

Old age psychiatry services have a significant role to play in the pandemic. Community teams are still conducting home visits to see the most vulnerable and offering regular review of those most isolated. However, the way we work has altered, with increasing use of virtual consultations, to enable us to interact effectively with our patients and their carers. These technologies have been particularly useful when completing medicolegal assessments for institutions such as the Court of Protection. The English Department of Health and Social Care guidance now includes and indeed recommends use of remote techniques such as telephone and video calls where appropriate to do so (Department of Health and Social Care, 2020). In our experience, assessments undertaken in this manner have been facilitated by carers, as in general, our patient group lacks the technology or the knowledge to make full use of these new platforms. Nonetheless, virtual consulting has been helpful and has allowed a fuller assessment than by telephone alone. Digital technology has also been used to bring families closer to their loved ones (e.g. using tablets for “FaceTime”) on ward where visiting has been suspended due to infection risks.

Community old age psychiatry teams are used to working flexibly as even within a generally underfunded National Health Service (NHS), mental health is even more under-resourced and that of older people is even more so (Royal College of Psychiatrists, 2018). This also means that we are able to utilize our experience of working with limited resources at a time when the nation is under significant constraints. In the UK, old age psychiatrists tend to work in a multidisciplinary way and have developed strong links with UK primary care and non-statutory stakeholders such as The Alzheimer’s Society and community rapid response teams. We provide in-reach to care homes and the networks developed with partners in primary care have strengthened during a time of national need to enable support to the care home population. Professor Helen Killaspy noted in March that “at present the COVID-19 case reporting processes do not include the level of detail that would inform the relative prevalence of suspected and confirmed cases and deaths amongst groups of people with different types of mental health problem” (Killapsy, 2020).

We have found that carers, both formal and informal, particularly value the advice and support offered in a unique situation where they feel very isolated and otherwise unsupported. An important and valuable aspect of the role of old age mental health services is to shine a light on the forgotten victims (and heroes) of the pandemic – the patients, family, and care staff in care homes and individual households, where provision of personal protective equipment and testing for COVID-19 was slow to materialise, and the death rates were initially not included in the official figures.

The sense in the UK is of greater caution in prescribing medication that could potentially precipitate respiratory depression, in particular benzodiazepines/depot antipsychotic. In our experience, Older Adult Liaison services are advising reducing/stopping depot to switch to orals whilst in hospital, to allow for greater flexibility in terms of dosing and reversibility as well. Conversely (anecdotally), more “anticipatory” prescribing appears to be occurring. This is probably a combination of pragmatism and wish to reduce risk – if a script (e.g. for antipsychotic) is provided earlier, the footfall will be less, as there will be less “toing and froing” between assessments and so less face-to-face contacts. The way that UK old age psychiatry works (borne out of limited resources), with strong partnerships between primary and secondary care, and good communication with patients and carers, lends itself to this strategy. However, the risks associated with this approach must be considered; sadly, more holistic and nuanced management is often precluded in an age of social distancing and need to manage risk safely. Some Community Mental Health Teams (CMHT) are using mobile technology such as Kardia (an electrocardiogram [ECG] mobile app) to support rapid initiation of anti-psychotics in patients who are assessed to need this. Again, the use of mobile apps reduces the footfall.

Monitoring of the physical parameters for antipsychotics has also become more pragmatic. For example, after consultation with Zaponex Treatment Access System (ZTAS) (a UK clozapine monitoring service), monitoring has been suspended for a patient on clozapine (with green results for over 30 years), to reduce risk of COVID-19 infection, having carefully considered the risks vs benefits of this strategy.

In the UK, London was initially the epicenter of COVID-19, and old age psychiatrists in the capital have rapidly reconfigured their services in response to the challenges that have arisen. We are used to advising on capacity, as well as considering ceilings of care and palliative management in patients with dementia. However, in this current situation, the nature of our work is shifting. Sadly, given the situation in general hospitals, patients who might previously have been moved if they developed COVID-19 may no longer have this option, and our inpatient wards have developed new protocols working together with colleagues in primary care and palliative care, taking into account the likely palliative care needs of our patients. It has been satisfying to recognize that we as old age psychiatrists have the expertise to provide excellent palliative care for our patients, having put into place many of the processes to do this effectively on our wards in pre COVID-19 times. Old age psychiatry wards already possessed careful documentation of capacity and resuscitation status and discussion around best interests of individuals admitted to the ward. In south-west London, best practice guidelines have been developed for providing palliative care on psychiatric wards using the resources available. Local universities are upskilling mental health staff with rapid intense training in physical health care (e.g. administering subcutaneous medications) and working with trust pharmacy to look at how best to alleviate distressing symptoms using what is available and what our ward teams are trained to use (i.e. avoiding syringe driver/intravenous medications).

There is an increased sense of collegiate working, with all specialties trying to work together for the greater good, and this can be seen in the way that trusts have developed initiatives that before the pandemic may have taken months, if not years to come to fruition, but in these more urgent times, have occurred in days. One such development was in South West London, where a new acute mental health ward seeing all ages (initially) was developed to alleviate the pressure on the local A+E departments and enable better use of resources. However, this was with careful oversight and use of a dedicated old age liaison psychiatrist within the service. Similarly, regional centers have developed multidisciplinary ethics committees which consider the ethical dilemmas thrown up by the pandemic. Nationally, community old age mental health services, freed from the shackles of the overwhelming administrative burden of recent years, have been able to focus on the most important issues. These include focusing on vulnerability and risk with appropriately targeted provision of support, rather than spurious key performance indicators.

The Royal College of Psychiatrists, with its attendant faculties, has provided guidelines on working together and has set up forums to share good practice (Royal College of Psychiatrists, 2020) and disseminate information about the new coronavirus legislation and proposed changes to the Mental Health Act. Alongside these formal channels, informal networks and communities of professional support have appeared on social media, again sharing useful experience at all levels: local, national, and international. All of these have been helpful in informing service provision at a time of national crisis and need to work sensitively within the constraints of rapidly changing legal frameworks (Essex Chambers, 2020).

COVID-19 has led to a wholesale shift in the working pattern of the NHS as a whole and of old age psychiatry as a specialty in the UK. There will undoubtedly be a legacy once lockdown is lifted. One huge concern within the specialty is that of an overwhelming increase in mental health need from older people who have had social isolation enforced due to quarantine and/or hospitalization. They may have had little access to the technology that has allowed younger members of society to alleviate their isolation to some extent. Given the limited number of old age psychiatrists and specialist old age mental health workers and the increasing proportion of older people in our society, this is, and should be, worrying. We are already seeing increased presentations of anxiety and low mood in our population (over 75s), who have had to suffer the greatest restrictions. This echoes the issues seen in this demographic during past pandemics, but we predict the effect size is likely to be larger given the scale of this pandemic.

Unfortunately, research programs have been paused/halted with potentially significant impact on conditions such as dementia and mental illness. This is likely to have long-term consequences on the development of new or better treatments for these conditions. Of course, the immediate and long-term psychiatric consequences of the pandemic and infection from COVID-19 will also need research, ideally via imaginative interdisciplinary collaboration (Holmes *et al.*, 2020).

Nonetheless, there are many positives that are also becoming apparent: change which was already occurring in the NHS and society has been accelerated, including increasing flexible working patterns and greater use of virtual/remote consulting via online platforms. This may lead to less domiciliary visiting in the future, but potentially more efficient use of resources. There has been an increase in interdisciplinary working: barriers between specialties and services breaking down with a greater willingness to work together in a flexible manner, for the greater good.

This has been echoed throughout society, with an unprecedented number of people volunteering to help more vulnerable members of society. Communities are rallying together and have set up medicine deliveries, emergency food parcels, online choirs, recipes, and mindfulness sessions and are offering support to family carers. Perhaps there is increased empathy for isolated older people who were already in a quarantine of sorts due to lack of mobility/frailty and limited income. UK society is also more appreciative of all workers in the NHS, but in particular those who work in intensive care/acute hospitals and those who work with older people, with national “clapping for carers” (literally providing a round of applause as a tangible way of showing their gratitude) at a set time each week. Despite the national shortage of old age psychiatrists and specialist older adult mental health workers, we are fortunate to have well-developed networks (both within our specialty and with other disciplines) and the ability (borne from experience of needing to function in the midst of longstanding relatively limited resources) to work flexibly, which has served the specialty well. There is also greater recognition of the need for expertise in mental health in society, with more high profile advocates such as the Duke and Duchess of Cambridge. During this pandemic, the need for expertise in older adults’ mental health has become apparent, and in the future, there may be greater demand for old age mental health services to be embedded within community networks, though it remains to be seen if the rhetoric will be followed by action on this front, given past depletion of the specialty (Warner, 2014).
